# pH-Responsive Artemisinin Derivatives and Lipid Nanoparticle Formulations Inhibit Growth of Breast Cancer Cells *In Vitro* and Induce Down-Regulation of HER Family Members

**DOI:** 10.1371/journal.pone.0059086

**Published:** 2013-03-14

**Authors:** Yitong J. Zhang, Byron Gallis, Michio Taya, Shusheng Wang, Rodney J. Y. Ho, Tomikazu Sasaki

**Affiliations:** 1 Department of Chemistry, University of Washington, Seattle, Washington, United States of America; 2 Department of Medicinal Chemistry, School of Pharmacy, University of Washington, Seattle, Washington, United States of America; 3 Department of Pharmaceutics, School of Pharmacy, University of Washington, Seattle, Washington, United States of America; Boston University Goldman School of Dental Medicine, United States of America

## Abstract

Artemisinin (ART) dimers show potent anti-proliferative activities against breast cancer cells. To facilitate their clinical development, novel pH-responsive artemisinin dimers were synthesized for liposomal nanoparticle formulations. A new ART dimer was designed to become increasingly water-soluble as pH declines. The new artemisinin dimer piperazine derivatives (ADPs) remained tightly associated with liposomal nanoparticles (NPs) at neutral pH but were efficiently released at acidic pH's that are known to exist within solid tumors and organelles such as endosomes and lysosomes. ADPs incorporated into nanoparticles down regulated the anti-apoptotic protein, survivin, and cyclin D1 when incubated at low concentrations with breast cancer cell lines. We demonstrate for the first time, for any ART derivative, that ADP NPs can down regulate the oncogenic protein HER2, and its counterpart, HER3 in a HER2+ cell line. We also show that the wild type epidermal growth factor receptor (EGFR or HER1) declines in a triple negative breast cancer (TNBC) cell line in response to ADP NPs. The declines in these proteins are achieved at concentrations of NP109 at or below 1 µM. Furthermore, the new artemisinin derivatives showed improved cell-proliferation inhibition effects compared to known dimer derivatives.

## Introduction

Artemisinin (ART), a natural product isolated from the plant *Artemisia annua*, was discovered in the early 1970s by Tu et al [1], [2]. ART and its derivatives, alone or in combined therapy, are the standards of care for all forms of malaria[3], [4]. ART-based anti-malarial drugs have excellent safety profiles [5], [6] while demonstrating extraordinary activity. Recently, Posner et al reported that malaria in a mouse model can be cured with a single dose of an ART-dimer derivative [7]. ART contains an endoperoxide bridge (R-O-O-R') that is required for its anti-malarial activity. Malaria parasites digest hemoglobin as a carbon source, and accumulate a large amount of iron [8]–[10]. When ART encounters an iron atom, the endoperoxide group breaks up, and forms free radicals. These carbon-based radicals, when formed within a malaria parasite, can lead to cellular damage and cell death.

Interestingly, cancer cells are also sensitive to ART due to their elevated iron uptake and metabolic activities [11]–[15]. Derivatives of ART have shown promising anticancer effects against multiple cell lines derived from various types of cancers [16]–[25], with dimeric and oligomeric derivatives showing greatly enhanced efficacy[18]–[21], [26]–[28]. ART derivatives induce apoptosis in human cancer cell lines [29] and simultaneously down-regulate proteins such as c-myc, cyclin D1, and survivin, which are known to be involved with oncogenesis, the cell cycle, and apoptotic resistance [30], [31] respectively. Both ART monomer and dimer derivatives have shown activity *in vivo* in mouse xenograft models [32], [33], especially for breast cancer models [34].

Despite the encouraging *in vitro* and animal model data, several key issues need to be addressed before further clinical development of ART derivatives as cancer chemotherapy can take place. ART derivatives, a class of sesquiterpene, generally possess poor aqueous solubility. Chemical approaches to solubilize the compound in an aqueous enironment have only been probed briefly, such as the development of artesunate[35], a water-soluble derivative. For dimers, however, the succinate ester analogue does not suffice at higher concentrations [28]. Alternative approaches, such as carrier conjugated ART derivatives, are also scarce in the literature, given the promising cytotoxicity results reported by numerous groups. Furthermore, the rapid clearance of the free drug molecules from blood circulation (artesunate <15 minutes)[36] makes these compounds unsuitable as treatments for cancer in free drug form.

The use of a nanoparticle (NP) carrier to incorporate or encapsulate the desired drug and deliver it to the target site *in vivo* to improve the bioavailability and pharmacokinetics of the drug molecules is no foreign concept today[37]. Among the multitude of nanoparticle systems being studied, liposomal nanoparticles represent a class of better developed delivery vehicles[38]. Both classical liposomes composed of only lipids and cholesterol, and stealth liposomes containing PEGylated lipids have been developed as anti-fungal, anti-cancer, anti-HIV, etc. therapies. Doxil^®^ is one example of a commercialized liposomal cancer chemotherapeutic.

Nanoparticles of size less than 200 nm are able to pass through the solid tumor microvasculature due to the enhanced permeability and retention (EPR) effect at these sites [39]. As the NPs accumulate, there forms a local microresevior of drugs for an enhanced biodistribution [40]. The accumulation, however, may not correlate to bioavailability when dug release at solid tumor sites into cells is inefficient.

While there may be multiple potential solutions to this problem, we sought a pH-dependent loading-release mechanism in our approach with ART-containing NPs. The working principle of such delivery systems relies on the acidified tumor microenvironment (pH = 6.5–7.0) compared to physiological pH of 7.4, and a further acidification to as low as pH 4.8 in the endosome/lysosome network after cellular uptake [41]–[43].

**Figure 1:**
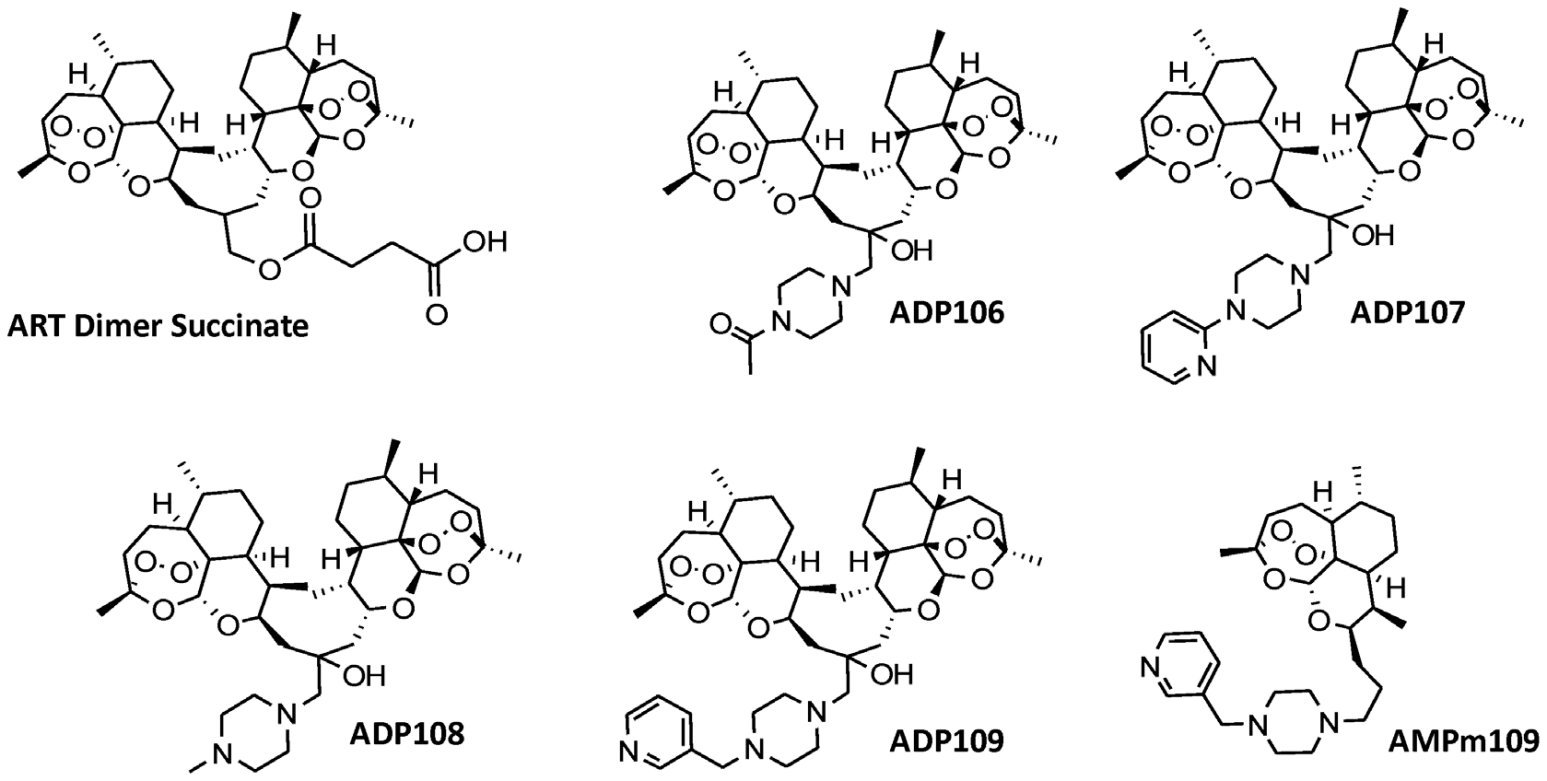
Structures of artemisinin dimer succinate, ADPs 106–109 and ADPm109 the monomer analogue of compound ADP109.

Here, we report the syntheses of four novel ART dimer piperazine conjugates (ADPs, **Fig. 1**) that show pH-responsive aqueous solubility profiles, as well as one of the first liposome nanoparticle formsulations for *in vitro* characterization. We also demonstrate that these nanoparticles down-regulate multiple proteins in two types of breast cancer cell lines that maintain and contribute to their malignant state.

## Methods

### Synthesis of Trioxane Isobutylene Dimer 2

Trioxane isobutylene dimer **2** was synthesized in two steps from artemisinin by following the procedure described by Posner et al [27].

### Synthesis of Bis-Trioxane Epoxide 3

*meta*-Chloroperbenzoic acid (mCPBA) was purified by dissolving 0.5 g of the commercial material (≤77%) in 5 mL of diethyl ether and extracting twice with 1 mL of 0.1 M potassium phosphate buffer (KH_2_PO_4_/K_2_HPO_4_) at pH 7.2.The ether layer was dried over Na_2_SO_4_ and the solvent was removed *in vacuo* to obtain pure mCPBA. To a 50 mL round bottom flask under N_2_ was charged 0.24 g (0.4 mmol) of **2** dissolved in 15 mL of dry dichloromethane (DCM). The solution was cooled to 0°C before 0.14 g (0.8 mmol, 2 eq.) of mCPBA, dissolved in 10 mL dry DCM was added dropwise under nitrogen. The reaction mixture was stirred at 0°C for 30 minutes before warming up to room temperature for additional 3 hours with stirring. The consumption of the reactant **2** was confirmed by TLC (30% ethylacetate (EA) in hexane (H)) before the reaction was quenched with a mixture of 4 mL saturated sodium bicarbonate (NaHCO_3_) and 4 mL 0.1 N disodium carbonate (Na_2_CO_3_). The reaction was stirred for 15 minutes. The organic layer was then extracted 3 times with 10 mL saturated NaHCO_3_, dried over Na_2_SO_4_, concentrated under reduced pressure to obtain the epoxide. The product was used for subsequent reaction without further purification. Analytical data matched that reported in literature [27].

### Synthesis of Bis-Trioxane Piperazine Conjugates (ADPs) 106–109

ADPs **106–109** were synthesized by using the same general procedure described as follows. In a 1 dram glass vial with a magnetic stir bar were placed ca. 60 mg (0.1 mmol) of **3** and 8.6 mg (0.1 mmol, 1 eq.) of lithium bromide and then 200 µL methanol:DCM (1∶5). To the solution, 0.2 mmol (2eq.) of piperazine derivatives were added to start the reaction. The vial was briefly purged under N_2_, capped and stirred at room temperature for 16–40 hours depending on the compound (see below). Upon consumption of **3** monitored by TLC, the reaction mixture was diluted with 2 mL of DCM, extracted 4 times with 1 mL 0.1 N Na_2_CO_3_, dried over Na_2_SO_4_. The solvent was then removed *in vacuo* to yield the crude products. Each product was purified by column chromatography. Detailed characterization of **ADP 106–109** available in Supporting Information.

### Egg Phosphatidylcholine 1-Bis-Trioxane-4-(3-pyidyl) methylpiperazine Lipsome (NP109) Preparation

The general procedure follows that of the previously published protocol[44]. **ADP109** was dissolved in chloroform to make a 10 mg/mL stock solution. The EPC solution was purchased at the concentration of 100 mg/ml. Aliquats of **ADP109** and EPC stock solutions were added to a screw-capped glass test tube, dried under a gentle stream of N_2_ followed by *in vacuo* overnight to form a thin film on the inner wall. The film was rehydrated with 0.9× PBS at 40°C for 10 minutes to give a liposome suspension at 20 mM lipid concentration. The mixture was then sonicated for 5 minutes 3 times to give a translucent suspension without observable particles, to afford the desired nanometer sized liposomes. **NP109** was diluted to 2 mM in a test tube for sizing measurements on Zetasizer 5000 (Malvern Instrument, Worcestershire, UK) with argon laser at 633.0 nm at room temperature. Liposome populations generally show narrow distribution (peak width less than 20 nm) with occasionally 1% of peak at ∼300 nm by intensity (but not visible by volume or number). Mean particle size reported in manuscript represents average and standard deviation calculated from number measurements of at least 3 sizing experiments.

### Loading and Release Efficiencies

For loading efficiency studies, liposome suspensions were placed into a dialysis tubing (MWCO 6000–8000 Da) and dialyzed against at least 1000× volume of 0.9× PBS buffer for 16 hours at room temperature. Equal volume aliquots of both dialyzed and undialyzed samples were collected in separate 5 mL glass test tubes, and extracted with 1 mL of spectroscopy-grade DCM 3 times. The combined organic layer was dried over Na_2_SO_4_. The solvent was then removed *in vacuo*. The dried solids were redissolved in acetonitrile (HPLC grade) for the UV absorbance measurement from 200 to 400 nm by DU 640 spectrophotometer (Beckman Culter, USA). More details see SI.

The percent loaded was calculated according to the following equation (**Eq. 1**): 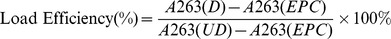



Where A_263_ (D) is the absorbance at 263 nm of the dialyzed sample, A_263_ (UD) is that of the undialyzed sample, and A_263_ (EPC) is that of EPC alone. Values used for calculation for a single experiment are the average of triplicate readings.

For the release efficiency studies 150 µL of 20 mM dialysis-purified liposome suspensions were placed in dialysis tubing and dialyzed against 500 mL of buffers of pH 7.6 PBS, pH 6 citrate and pH 4 citrate separately for 24 hours at room temperature. 30 µL of dialyzed samples were collected afterward for UV studies with the same workup procedure as that of the loading efficiency studies.

The percent released was calculated according to **Eq.2**: 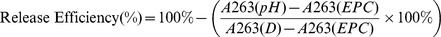



Where A_263_ (pH) is the absorbance at 263 nm of the sample dialyzed for 24 hours at various pH values, A_263_ (D) is that of the dialysis-purified sample, and A_263_ (EPC) is that of EPC alone. Values used for calculation for a single experiment are the average of triplicate readings.

*MTT Assays.* In a 96 well plate was seeded ca. 2000 cells/well for BT47, 7000 cells/well for MDA-MB-231 or 5000 cells/well for MDA-MB-468 and SKBR3 and incubated until fully adhered (20–40 hours) at 5% CO_2_ in DMEM containing 10% FBS (Complete Medium) at 37°C. Serial dilutions of ADP stock solutions at 20 mM in DMSO were made to 8 appropriate concentrations ranging from 1 nM to100 µM, depending on the specific compound, in Complete Medium with 1% DMSO. 200 µL of the compound containing medium were added to each well after removal of the original exhausted medium. NP concentrations were calculated assuming 100% loading for both formulations. Three wells were run in parallel for any given compound and concentration. The negative control was 1% DMSO containing Complete Medium and positive control employed 100 µM ART dimer succinate. The cells were incubated with the drugs for 48 hours at 37°C before the medium was replaced with 90 µl of fresh Complete Medium plus 10 µl of MTT solution at 5 mg/mL concentration and incubated further for 4 hours. At the end of incubation time, exhausted medium was gently removed and the purple formazan crystals were dissolved in 50 µl of DMSO, incubated for 10 minutes before the absorbance at 570 nm was read on microplate reader model 680 (Bio-Rad, California, USA).

### Western Blots

Western blots were performed as described [29]. Primary antibodies used in Western blots were rabbit monoclonal antibodies (rabmabs) to cyclin D1, HER2, and HER3 from Epitomics, Inc (Burlingame, CA), rabmabs to survivin and HER1 from Cell Signaling Technology, Inc (Danvers, MA) and mouse mab to actin, clone AC-15 (Sigma, St. Louis, MO). Three independent experiments were performed for all Western blots.

### Protein Assay Cell Culture

BT474 and MDA-MB-231 cells were obtained from the American Type Culture Collection (Manassas, VA) and maintained in DMEM with L-glutamine (Invitrogen Corporation) and supplemented with 10% fetal bovine serum (Atlanta Biologicals, Lawrenceville, GA) and penicillin/streptomycin. Liposomes and drug containing NPs were suspended in PBS and diluted into cell culture media.

Detailed experimental descriptions (**Text S1**) and additional figures (**Figures S1, S2, S3, S4, S5, S6, S7, S8, S9, and S10**) in Supporting Information.

## Results

### Synthesis of pH-responsive Artemisinin Dimer Piperazine Conjugates (ADPs)

**Figure 2:**
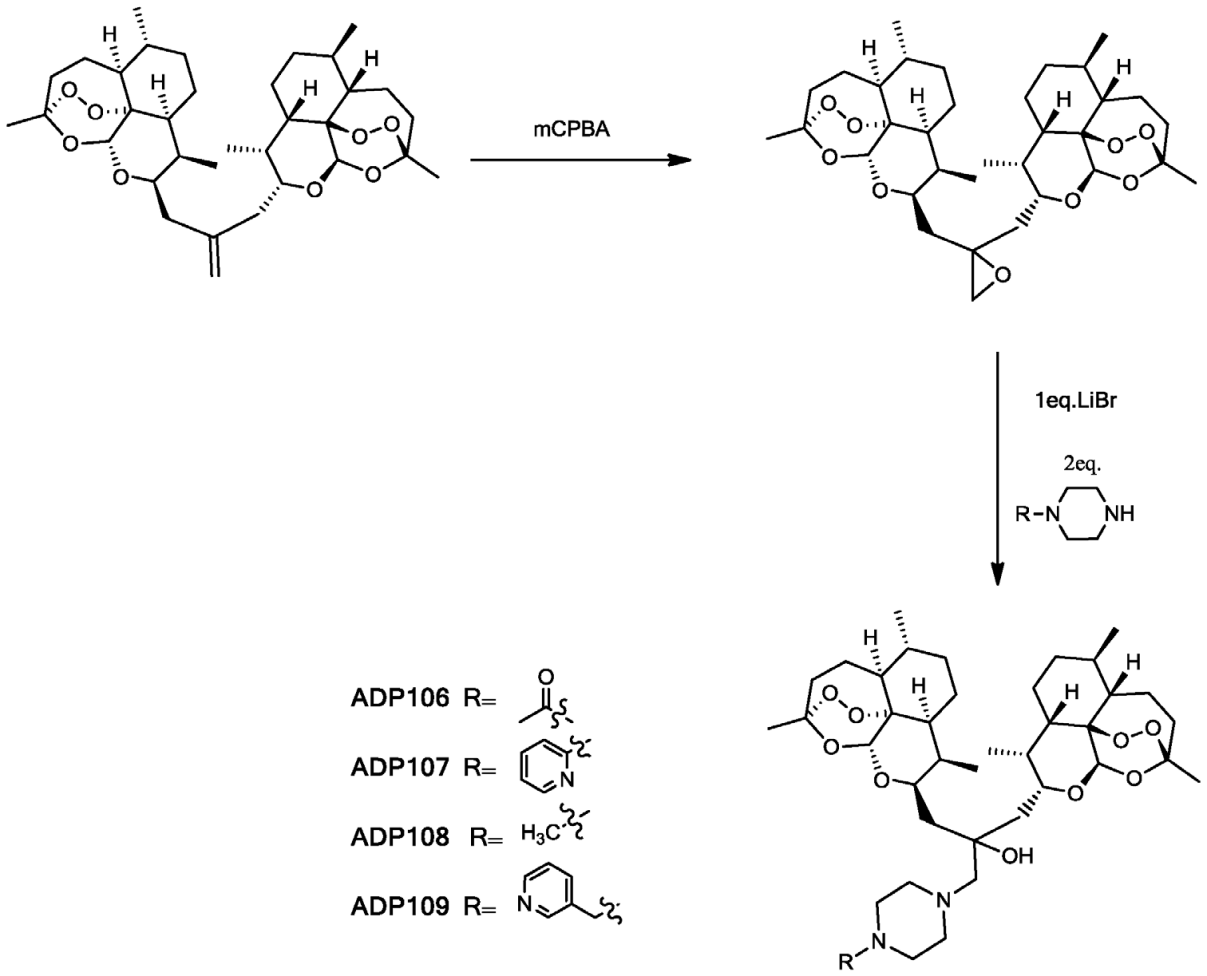
Synthesis of ADPs 106–109.

In synthesis of the ADPs (**Fig. 2**), ART Isobutylene Dimer **2** was prepared according to the literature [27]. The double bond in **2** was then epoxidized with *meta*-chloroperbenzoic acid (mCPBA) to give **3** for the subsequent epoxide-opening reaction. Compound **3** and corresponding piperazine were reacted in the presence of lithium bromide at room temperature, affording final **ADP106-109** in 53–81% yield (detailed synthesis see Supporting Information). The pH-responsive moieties of the ADPs draw analogy from indinavir, a potent HIV drug, which demonstrated pH-responsive properties in lipid nanoparticle formulations [45], [46]. An ART-monomer piperazine conjugate (**AMPm109)** was also synthesized for comparison in characterizations. For the monomer derivative, the initial approach with epoxide ring-opening reaction produced inseparable diastereomers. Therefore, we utilized reductive amination of ART 10-β-propanal **9** (details see Supporting Information) to circumvent the complications with the hydroxyl group in the arm. The effect of the hydroxyl group was confirmed to be minimal by MTT assay (data not shown).

**ADP106–109** and **AMPm109** were characterized by ^1^H-NMR and MALDI-TOF (**Fig. S1, S2, S3, S4**), and structures are shown in **Fig. 1**. The solubilities of the compounds were estimated by visual turbidity test in phosphate/citrate buffer at various pH values (**Table S1** in Text S1). Of the ADPs, **ADP109** showed the most desirable solubility profile with 0.034 mM at pH 7.4, 0.19 mM at pH 6 and 1.5 mM at pH 4. **ADP109** was thus chosen as the lead compound for nanoparticle formulation (**NP109**).

### Nanoparticle Formulation with ADP109 and AMPm109

L-α-Phosphatidylcholine extracted from eggs (EPC) was used to construct the liposome formulation for our initial showcase formulations **NP109** (EPC + **ADP109**) and **NPm109** (EPC + **AMPm109**). We prepared the liposome nanoparticles with the liquid-hydration-sonication method previously described [45], [46]. Briefly, a 10-to-1 ratio of EPC and **ADP109**, both in chloroform, were mixed and dried down to form a thin film before rehydrated in 0.9× phosphate buffered saline (PBS) and sonicated to give a suspension of the nanoparticles at a concentration of 20 mM of lipid. Photon correlation spectroscopy showed that the sonicated suspension contained mono-dispersed nanoparticles with the size of 70 nm (±20 nm) in diameter for **NP109** and 50 nm (±20 nm) for the ART monomer analogue **NPm109**.

Loading efficiencies of ADP-NPs were determined by comparing the UV absorbance values measured at 263 nm for the dialyzed and original samples (**Eq. 1**). An average of three independent experiments gave a loading efficiency of 91% (±9%) for NP109 and 63% (±13%) for **NPm109** (**Table 1**). The release of the drug from NPs was determined by equilibrium dialysis for 24 hours against buffers at various pH values and then comparing the UV absorbance values at 263 nm to that of time zero. **NP109** retained 99% of the bound drug at pH 7.6, released 14% at pH 6 and 52% at pH 4 after 24 hours. In contrast, NPm109 lost 13%, 91% and 96% of **AMPm109** at pH 7.6, 6 and 4 respectively (Spectra **Fig. S7, S8**). The loading and release profiles of both compounds effectively demonstrated the dependence on the environmental pH that was originally designed into the derivatives.

**Table 1:** Summary of loading and release efficiencies of the NPs.

Loading and Release Efficiencies of NP109 and NPm109				
	% Association	% Released (24 hrs)		
		pH 7.6	pH 6	pH 4
**NP109**	**91 (±9)**	**1 (±4)**	**14 (±14)**	**52 (±14)**
**NPm109**	**63 (±13)**	**13 (±3)**	**91 (±10)**	**96 (±7)**

Values represent an average and standard deviation of three independent experiments read at λ = 263 nm.

### *In vitro* Cytotoxicity Study

Next, MTT assays were performed to evaluate the ability of **ADP106–109** to inhibit cell growth on BT474 (HER2+), and MDA-MB-231, (“triple-negative” or TNBC) human breast tumor cell lines (**Fig. S9 and S10**). **Table 2** shows the IC_50_ values. (Other MTT data see **Table S2** in Text S1) All the compounds except for **AMPm109** showed sub-micromolar IC_50_ values on BT474 cells, consistent with high antiproliferative effects reported in the literature for other artemisinin dimers. Monomeric artemisinin derivative, **AMPm109**, showed a comparable activity to that of artesunate (data not shown). The nanoparticle formulations retained the potency of the free drug, confirming that the drugs are efficiently loaded and released from liposomes.

**Table 2:** Summary of IC_50_ values calculated from MTT assays of ADPs and NPs on BT474 and MDA-MB-231 cells.

Cell Toxicity MTT Assay of ADPs		
	IC50 (µM)	
	BT474 (± S.D.)	MDA-MB231 (± S.D.)
**ART Dimer Succ.**	**0.39 (±0.27)**	**32 (±4)**
**ADP106**	**0.06 (±0.03)**	**8 (±3)**
**ADP107**	**0.022 (±0.009)**	**5 (±2)**
**ADP108**	**0.11 (±0.02)**	**3.3 (±0.7)**
**ADP109**	**0.07(±0.01)**	**10 (±3)**
**AMPm109**	**1.3 (±0.8)**	>>100*
**NP109**	**0.08 (±0.01)**	**7 (±2)**
**NPm109**	**1.3 (±0.4)**	>>20*

Values represent average (±SD) calculated from three independent experiments. *Exceeded maximum concentration of assay.

### NP109 down regulates proteins which support neoplasia in two breast cancer cell lines

**Figure 3:**
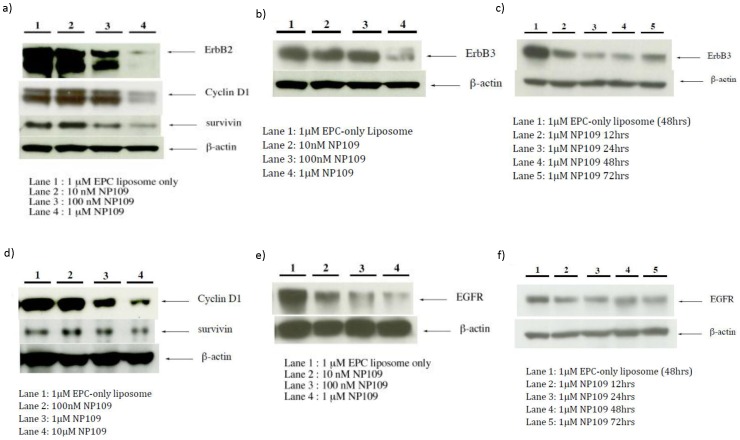
Effects of NP109 on the expression of selected proteins involved in cell proliferation, cell cycling, and apoptosis in BT474 (a–c) and MDA-MB-231 cells (d–f).

A closer look at the biochemical responses of BT474 and MDA-MB-231cells incubated with NP109 at nanomolar and low micromolar concentrations showed marked declines of both survivin, which causes resistance to apoptosis[47], and cyclin D1, a protein integral to cell replication[48]. Dose-response experiments showed that both survivin and cyclin D1 were decreased in the presence of 100 nM to 1 µM **NP109** in BT474 cells (**Figure 3a**) and 1 µM **NP109** in MDA-MB-231 cells (**Figure 3d**).

Since survivin levels have been shown to be coupled to HER2 expression [47], we also observed that HER2 levels declined when BT474 cells were incubated with 100 nM–1 µM **NP109** (**Figure 3a**). In addition, the level of another mutated form of the epidermal growth factor receptor, ErbB3 (HER3), frequently expressed in HER2+ cell lines, was shown to decrease in BT474 cells in dose response (**Figure 3b**) and time course (**Figure 3c**) experiments with **NP109**. Epidermal growth factor receptor, EGFR (HER1), over-expressed in triple negative breast cancers [49], was down-regulated in MDA-MB-231 cells by **NP109** in the dose response (**Figure 3e**) and time course (**Figure 3f**) studies. In summary, we have shown that **NP109** was able to induce decline in the levels of the same proteins, survivin and cyclin D1, as well as over-expressed and mutated forms of EGFR in BT474 (HER2+) and MDA-MB-231 (EGFR) cells.

When the biochemical effects of **NP109** were determined for an additional HER2+ cell line, SKBR3, and another TNBC cell line, MDA-MB-468, survivin was strongly down regulated in both cell lines, but neither HER2/HER3 levels were altered in SKBR3 nor EGF levels changed in MDA-MB-468 cells (data not shown). Cyclin D1 levels were not examined.

## Discussion

The ART Dimer piperazine conjugates (ADPs) designed here were synthesized using lithium bromide (LiBr) as a mild Lewis acid [50] for the epoxide ring opening reaction. This method allowed incorporation of the pH-responsive piperazine moiety while avoiding use of heavy metal. The novel ADPs preserved the active core of the artemisinin dimer while introducing multiple protonation sites to increase aqueous solubility as pH lowers from 7.4 under physiological conditions to below 5 in lysosomes [51].

In the absence of LiBr, the epoxide opening reaction hardly gave any product after overnight conditions. Reactions using tin(II) trifluoromethanesulfonate at both room temperature and under reflux conditions resulted in large amounts of decomposition and little desired products. The lower loading efficiency for **AMPm109** is likely due to its higher solubility at neutral pH.

The ADP-NPs described in this manuscript is the first liposomal nanoparticle formulation of artemisinin dimer derivatives, to the best of our knowledge. We were able to demonstrate efficient incorporation of the novel artemisinin derivatives **ADP109** and **AMPm109** at physiological pH and a pH-dependent release of the incorporated drugs, according to design. The data attest to the potential of the liposome-nanoparticle approach for selective *in vivo* delivery of the ADPs to tumor cells.

This study also examined selected biochemical changes in a HER2+ (BT474) and a TNBC (MDA-MB-231) cell line in response to an ART derivative-containing NP. Both survivin and cyclin D1 were down-regulated in the two cell lines as they are in prostate cancer cell lines [30] by a different ART derivative. Other investigators have shown that ART-derivative-induced declines in survivin protein levels in cancer cell lines are accompanied by declines in survivin mRNA [52], [53]. Survivin is an anti-apoptotic protein that is expressed in the majority of tumors in most human cancers [54] and is correlated with poor prognosis in breast cancers [55]. It is, however, not detected in terminally differential healthy tissues. This differential expression has made it a target for potential cancer therapy [56], [57]. Among the cyclin isoforms, cyclin D1 over-expression is most frequently associated with human cancers [58]. Since cyclin D1 is an important regulator of G1 to S-phase during the cell cycle, ART induced degradation of this protein may be sufficient to arrest cell growth in some cancers [59].

Known as a marker, the over-expression of HER2, a member of the epidermal growth factor receptor tyrosine kinase family, occurs in 25% of breast cancers, predict for poor clinical outcomes, and resistance to chemotherapies [60]–[62]. While it has no activating ligand, HER2 is activated by homo-and hetero-dimerization with wild type EGFR (HER1), HER3, or HER4. The poor prognosis for patients with tumors over-expressing HER2 is believed to be due to activation of the PI3-k/Akt family of kinases [63]. HER2 preferentially heterodimerizes with HER3, which while having no tyrosine kinase activity on its own contains six PI3-kinase docking sites, making the HER2/HER3 heterodimer a potent activator of this pathway [64]. The unique roles and properties of HER3 cause resistance to chemotherapies [61], [65]. Recent studies conclude that only combined blockade of HER2 and HER3 will be effective in treatment of HER2-mediated breast cancers [61], [62], [65]. Furthermore, the EGF receptor is over-expressed in 50% or more of triple negative breast cancers [49]. Presently, it is unclear what and how important a role EGFR amplification plays in the etiology of TNBC.

We have shown in our studies that **NP109** caused a decline of EGFR level in the TNBC cell line MDA-MB-231 and near elimination of HER2 level in the HER2+ cell line BT474. The ability of nanoparticles containing a pH sensitive ART derivative to down-regulate HER1, HER2, and HER3, as well as other proteins linked to neoplasia, suggests that this approach has real therapeutic potential for treatment of specific breast cancer subtypes.

In conclusion, we designed and synthesized a series of pH-responsive artemisinin dimers and successfully incorporated a candidate derivative into liposomal nanoparticles without significantly compromising its *in vitro* efficacy. Our studies clearly show that ADP-NPs elicit a unique set of biological responses from breast cancer cells to ultimately induce cell death, potentially opening up new avenues for treatments of breast cancer. The optimization of the lipid composition for these NPs is ongoing in our lab using animal models to evaluate the *in vivo* characteristics of ADP-NPs for further clinical development.

## Supporting Information

Figure S1 **^1^H-NMR (a) and MALDI-TOF (b) of ADP106.**
(TIF)

Figure S2 **1H-NMR (a) and MALDI-TOF (b) of ADP107.**
(TIF)

Figure S3 **1H-NMR (a) and MALDI-TOF (b) of ADP108.**
(TIF)

Figure S4 **1H-NMR (a) and MALDI-TOF (b) of ADP109.**
(TIF)

Figure S5 **1H-NMR (a) and MALDI-TOF (b) of AMPm109.**
(TIF)

Figure S6 **Design of the pH-sensitive ART Dimer Piperazine conjugate (a), and schematic diagram of the designed pH responsive ADPs in liposome nanoparticles (b).**
(TIF)

Figure S7 **Typical UV spectra overlay of dialyzed and undialyzed (a) and retention of drug after 24-hrs dialysis in buffers at various pH values (b) of NP109**. Spectra (a) shown is the average and (b) the average and standard deviation graphed from three parallel readings of one experiment.
(TIF)

Figure S8 **Typical UV spectra overlay of dialyzed and undialyzed (a) and retention of drug after 24-hrs dialysis in buffers at various pH values (b) of NPm109.** Spectra (a) shown is the average and (b) the average and standard deviation graphed from three parallel readings of one experiment.
(TIF)

Figure S9 **Overlay of typical cell viability data calculated from absorbance values at 570 nm of MTT assays of BT474 (a) and MDA-MB231(b) cells incubated with ADPs for 48 hrs with 10%FBS in DMEM at 37°C, 5%CO_2_ at various concentrations.** IC_50_ values see Table 2.
(TIF)

Figure S10 **Overlay of typical cell viability data calculated from absorbance values at 570 nm of MTT assays of BT474 cells incubated with EPC empty liposome, free drug ADP109, NP109 (a), or EPC empty liposome, free drug AMPm109, NPm109 (b) for 48 hrs with 10%FBS in DMEM at 37°C, 5%CO_2_ at various concentrations.** IC_50_ values see Table 2.
(TIF)

Text S1 **Supporting Information Text.**
(DOC)
