# Supplementary material for: pH-Responsive Artemisinin Derivatives and Lipid Nanoparticle Formulations Inhibit Growth of Breast Cancer Cells In Vitro and Induce Down-Regulation of HER Family Members
Source: PLoS One. 2013 Mar 14;8(3):e59086. doi: 10.1371/journal.pone.0059086 (PMC3597601; doi:10.1371/journal.pone.0059086)
Supplement: Text S1 — Supporting Information Text. (DOC) [file pone.0059086.s011.doc]

Supporting Information for

**3**

pH-Responsive Artemisinin Derivatives and Lipid Nanoparticle Formulations exhibit Growth Inhibition of Breast Cancer Cells *in vitro*

Yitong J. Zhang^†^, Byron Gallis^‡^, Michio Taya^†^, Shusheng Wang^†^, Rodney J.Y. Ho^§^, Tomikazu Sasaki^*†^

^†^Department of Chemistry, University of Washington, Seattle, Washington, 98195, United States

^‡^Department of Medicinal Chemistry and ^§^Department of Pharmaceutics, School of Pharmacy, University of Washington, Seattle, Washington, 98195, United States

**Methods**

**Materials** Artemisinin was purchased from Shaanxi Sciphar Hi-Tech Industry Co., LDT (Xi’an, China).Lithium Bromide, 1-acetylpiperazine, 1-(2-pyridyl)piperazine, 1-methylpiperazine were purchased from TCI America (Portland, OR), and 1-(3-pyridyl)methylpiperazine from Oakwood Products, Inc. (West Columbia, SC). All other chemicals and solvents used for synthesis were purchased from Sigma Aldrich (MO, USA) and were used without purification unless specified otherwise. L-α-phosphatidylcholine isolated from eggs (EPC) was purchased from Avanti Polar Lipids (Alabaster, AL). 10X PBS was purchase from Invitrogen (Eugene, OR).

Nuclear Magnetic Resonance(NMR) of compounds were recorded on Bruker’s AVANCE AV 500 MHz in CDCl_3_. MALDI-TOF mass spectra were recorded on a Bruker Autoflex II using 2,5-dihydroxy benzoic acid(DHB) and α-cyano-4-hydroxycinnamic acid (α-cyano) (2:1) matrix.

Cell cultures were generous gifts from Dr. N. Singh (MDA-MB-231) and Dr. B. Gallis (BT 474) for MTT assays. Thiazolyl blue tetrazolium bromide (MTT) was purchased from Sigma Aldrich (St. Louis, MO). Fetal bovine serum (FBS) and 0.25% trypsin-EDTA with phenol red were purchased from Invitrogen (Eugene, OR), and Dulbecco’s Modified Eagle’s Medium - high glucose (DMEM) and Dulbecco’s Phosphate Buffered Saline (PBS) were from Sigma Aldrich (St.Louis, MO).

The following antibodies were used for Western blotting: actin (clone 15) from Sigma-Aldrich (St. Louis, MO), survivin and epidermal growth factor receptor (EGFR) from Cell Signaling, Inc (Danvers, MA), HER2, HER3, and cyclin D1 from Epitomic, Inc (Burlingame, CA). Western blotting was performed as previously described^5^

**Synthesis of Trioxane Isobutylene Dimer** **2**. Trioxane isobutylene dimer **2** was synthesized in two steps from artemisinin by following the procedure described by Posner et al^1^.

**3**

**2**

**3**

**Synthesis of Bis-Trioxane Epoxide 3**. *meta*-Chloroperbenzoic acid (mCPBA) was purified by dissolving 0.5g of the commercial material (≤77%) in 5mL of diethyl ether and extracting twice with 1mL of 0.1M potassium phosphate buffer (KH_2_PO_4_/K_2_HPO_4_) at pH 7.2.The ether layer was dried over Na_2_SO_4_ and the solvent was removed *in vacuo* to obtain pure mCPBA.

To a 50mL round bottom flask under N_2_ was charged 0.24g (0.4mmol) of **2** dissolved in 15mL of dry dichloromethane (DCM). The solution was cooled to 0°C before 0.14g (0.8mmol, 2 eq.) of mCPBA, dissolved in 10mL dry DCM was added dropwise under nitrogen. The reaction mixture was stirred at 0°C for 30 minutes before warming up to room temperature for additional 3 hours with stirring. The consumption of the reactant **2** was confirmed by TLC (30% ethylacetate ( EA) in hexane (H)) before the reaction was quenched with a mixture of 4mL saturated sodium bicarbonate (NaHCO_3_) and 4mL 0.1N disodium carbonate (Na_2_CO_3_). The reaction was stirred for 15 minutes. The organic layer was then extracted 3 times with 10mL saturated NaHCO_3_, dried over Na_2_SO_4_, concentrated under reduced pressure to obtain the epoxide. The product was used for subsequent reaction without further purification. Analytical data matched that reported in literature ^1^.

**Synthesis of Bis-Trioxane Piperazine Conjugates (ADPs) 106-109**. ADPs **106-109** were synthesized by using the same general procedure described as follows. In a 1 dram glass vial with a magnetic stir bar were placed ca. 60mg (0.1mmol) of **3** and 8.6mg (0.1mmol, 1 eq.) of lithium bromide and then 200μL methanol:DCM (1:5). To the solution, 0.2mmol (2eq.) of piperazine derivatives were added to start the reaction. The vial was briefly purged under N_2_, capped and stirred at room temperature for 16-40 hours depending on the compound (see below). Upon consumption of **3** monitored by TLC, the reaction mixture was diluted with 2mL of DCM, extracted 4 times with 1mL 0.1N Na_2_CO_3_, dried over Na_2_SO_4_. The solvent was then removed *in vacuo* to yield the crude products. Each product was purified by column chromatography as shown below.

**1-Bis-Trioxane-4-acetylpiperazine (ADP106).** 21.5μL of 1-acetylpiperazine was added to the reaction mixture. The reaction was run for 16 hours before quenching. The crude product was subjected to flash chromatography with first 50% EA in H, then 5% aqueous ammonium hydroxide(NH_4_OH)-methanol (1:1) in DCM to give **ADP 106** as a white solid (39mg, 0.053mmol, 53%). ^1^H NMR (500 MHz, CDCl_3_) δ (ppm) 5.46 (s, 1H), 5.33 (s, 1H), 4.60 – 4.50 (m, 1H), 4.35 (dd, *J* = 11.1, 6.0 Hz, 1H), 3.54 (d, *J* = 30.7 Hz, 2H), 3.44 – 3.31 (m, 2H), 2.76 – 2.47 (m, 8H), 2.29 (ddd, *J* = 14.4, 9.5, 5.0 Hz, 2H), 2.13 (dd, *J* = 14.6, 11.8 Hz, 2H), 2.05 (s, 3H), 2.02 – 1.96 (m, 2H), 1.96 – 1.82 (m, 4H), 1.84 – 1.71 (m, 4H), 1.67 – 1.54 (m, 4H), 1.45 – 1.16 (m, 14H, including a doublet at 1.36, *J* = 5 Hz), 0.94 (dd, *J* = 5.6, 1.6 Hz, 6H), 0.95-0.86 (m, 2H), 0.88 (dd, *J* = 15.9, 8.0 Hz, 6H). MALDI-TOF *m/z* calcd for C_40_H_64_N_2_O_10_ 732.46 (M^+^), found, 733.565 ([M+H]^+^).

**1-Bis-Trioxane-4-(2-pyridyl)piperazine (ADP107).** 30.5μL of 1-(2pyridyl)piperazine was added to the reaction mixture. The reaction was run for 20 hours before quenching. The crude product was subjected to flash chromatography with gradient mobile system from 30% to 50% EA in H to give **ADP107** as a white solid (62mg, 0.081mmol, 81%). ^1^H NMR (500 MHz, CDCl_3_) δ (ppm) 8.19 – 8.15 (m, 1H), 7.45 (ddd, *J* = 8.7, 7.2, 2.0 Hz, 1H), 6.63 (d, *J* = 8.6 Hz, 1H), 6.60 – 6.56 (m, 1H), 5.49 (s, 0H), 5.37 (s,1H), 4.57 – 4.50 (m, 1H), 4.47 (d, *J* = 4.9 Hz, 1H), 3.48 (s, 4H), 2.90 – 2.82 (m, 2H), 2.70 – 2.65 (m, 2H), 2.64 – 2.56 (m, 2H), 2.76-2.56 (m, 2H), 2.31 (td, *J* = 14.0, 3.8 Hz, 2H), 2.08 (dd, *J* = 22.4, 10.8 Hz, 1H), 2.01 (d, *J* = 14.4 Hz, 2H), 1.95 – 1.75 (m, 8H), 1.68 – 1.57 (m, 4H), 1.45 – 1.19 (m, 14H, including a doublet at 1.36, *J* = 15.0 Hz), 0.95 (dd, *J* = 13.1, 6.2 Hz, 6H), 0.90 (dd, *J* = 7.5, 3.5 Hz, 6H), 0.97-0.85, (m, 2H). MALDI-TOF *m/z* calcd for C_43_H_65_N_3_O_9_ 767.49 (M^+^), found, 768.454 ([M+H]^+^).

**1-Bis-Trioxane-4-methylpiperazine (ADP108).** 22.2μL of 1-methylpiperazine was added to the reaction mixture. The reaction was run for 30 hours before quenching. The crude product was subjected to flash chromatography with first 25% EA in H, then 25% methanol in DCM to give **ADP108** as a white solid (50mg, 0.071mmol, 71%). ^1^H NMR (500 MHz, CDCl_3_) δ (ppm) 5.49 (s, 1H), 5.36 (s, 1H), 4.49 (d, *J* = 9.1 Hz, 2H), 2.68 (d, *J* = 13.5 Hz, 1H), 2.60 (dd, *J* = 11.4, 6.4 Hz, 2H), 2.54 (d, *J* = 13.4 Hz, 1H), 2.37 – 2.27 (m, 2H), 2.25 (s, 3H), 1.94 (ddd, *J* = 29.1, 22.9, 13.2 Hz, 8H), 1.81 – 1.71 (m, 4H), 1.69 – 1.54 (m, 12H), 1.45 – 1.17 (m, 14H, including a doublet at 1.39, *J* = 18.7 Hz), 0.98 – 0.95 (m, 6H), 0.91 – 0.88 (m, 6H), 0.98-0.89 (m, 2H). MALDI-TOF *m/z* calcd for C_39_H_64_N_2_O_9_ 704.46 (M^+^), found, 705.430 ([M+H]^+^).

**1-Bis-Trioxane-4-(3-pyidyl)methylpiperazine (ADP109).** 33.5μL of 1-(3-pyridyl)methyl-piperazine was added to the reaction mixture. The reaction was run for 40 hours before quenching. The crude product was subjected to flash chromatography with first 30% EA in H, then 5% aqueous ammonium hydroxide(NH_4_OH)-methanol (1:1) in DCM to give **ADP109** as a white solid (56mg, 0.071mmol, 71%). ^1^H NMR (500 MHz, CDCl_3_) δ (ppm) 8.50 (m, 2H), 7.67 (m, 1H), 7.24 (m, 1H), 5.47 (s, 1H), 5.35 (s, 1H), 4.47 (d, *J* = 37.4 Hz, 2H), 3.47 (s, 2H), 2.84 – 2.21 (m, 10H), 2.14 – 1.55 (m, 16H), 1.52 – 1.16 (m, 14H, including a doublet at 1.37, *J* = 13.0Hz), 0.96 – 0.94 (m, 6H), 0.88 – 0.86 (m, 6H), 1.06-0.82 (m, 2H). MALDI-TOF *m/z* calcd for C_44_H_67_N_3_O_9_ 781.49 (M^+^), found, 782.627 ([M+H]^+^).

**Synthesis of 10β-[(1-(3-pyidyl)methylpiperazyl)propyl]deoxoartemisinin (AMPm109)**. Compound **9** was synthesized according to the literature^2-3^. 69mg (0.2mmol) of **9** was dissolved in 1mL of dry DCM:methanol (1:1) in a 10mL 2-neck round bottom flask (Flask A) under N_2_ atmosphere and cooled to 0°C. In a separate 10mL round bottom flask, 28mg (0.44mmol, 2.2 eq.) of sodium cyanoborohydride was dissolved in 1mL of dry DCM:methanol (1:1) under N_2_ before transferred to Flask A. 38μL (0.2mmol, 1 eq.) of 1-(3-pyridyl)methylpiperazine was added to the reaction flask (A). The reaction mixture was warmed up to room temperature and stirred for 1 hour. TLC (30% EA in H) was used to check the consumption of **9** before 1mL of deionized water was added dropwise at 0°C to quench the reaction. The emulsion was then stirred for additional 30 minutes before organic solvents were removed under reduced pressure. The residue was redissolved in 2mL of DCM and extracted with 1mL of deionized water. The aqueous layer was extracted 3 times with 2mL DCM. The combined organic layer was then extracted with 5mL 0.1N Na_2_CO_3_ twice. The organic layer was dried over Na_2_SO_4_ and concentrated under reduced pressure. The product was purified by flash chromatography with 30% EA in H followed by 30% MeOH in DCM to give **AMPm109** as a white solid (20.3mg, 0.042mmol, 20%). ^1^H NMR (500 MHz, CDCl_3_) δ (ppm) 8.60 – 8.48 (m, 2H), 7.68 (d, *J* = 7.7 Hz, 1H), 7.34 – 7.21 (m, 1H), 5.31 (s, 1H), 4.16 (dd, *J* = 8.7, 4.9 Hz, 1H), 3.54 (s, 2H), 2.75 – 2.27 (m, 13H), 2.08 – 1.99 (m, 1H), 1.97 – 1.75 (m, 3H), 1.66-1.52 (m Hz, 4H), 1.49 – 1.21 (m, 7H, including a singlet at 1.42), 0.98 (d, *J* = 6.0 Hz, 3H), 0.87 (d, *J* = 7.5 Hz, 3H), 0.98-0.86 (m, 1H). MALDI-TOF *m/z* calcd for C_28_H_43_N_3_O_4_ 485.33 (M^+^), found, 786.463 ([M+H]^+^).

**Aqueous Solubility Test**. Buffers at pH 7.4, 6 and 4 were prepared from titration of 0.2M disodium hydrogen phosphate (Na_2_HPO_4_) with 0.1M citric acid solution to the desired pH without addition of other salts.

In five labeled 1.5mL eppendorf tubes was measured out 1mg of compounds **ADP106-109, AMPm109** and dissolved in 50μL of DMSO to make the stock solutions. 5μL of each of the stock solutions was pipetted into 5 separate small glass test tubes. 95μL of buffer was added to the test tubes, and mixture was vortexed for 10 seconds. The mixtures in the test tubes were allowed to settle and were observed for undissolved solids. If solids remained, 5μL DMSO and 95μL buffer were then added and process repeated until the mixture becomes a clear solution with no observable solid particles. For compounds **ADP106-108**, the process was stopped when there are still considerable amounts of particles in mixture after a total of 3mL of liquid were added.

**Egg Phosphatidylcholine-1-Bis-Trioxane-4-(3-pyidyl)methylpiperazine Lipsome (NP109)** **Preparation.** General procedure follows that of previously published protocol^4^. Compound **7** was dissolved in chloroform to make a 10mg/mL stock solution. The EPC solution was purchased at the concentration of100mg/ml. 31.3μL of **7**stock solution and 30.4μL of EPC solution were added to a screw-capped glass test tube, dried under a gentle stream of N_2_ followed by *in vacuo* to form a thin film on the inner wall. The film was rehydrated with 200μL of 0.9X PBS at 40°C for 10 minutes to give a liposome suspension at 20mM lipid concentration. The mixture was then sonicated for 5 minutes 3 times to give a translucent suspension without observable particles, to afford the desired nanometer sized liposomes. 50μL of the liposome suspension was diluted with 450μL of 0.9X PBS in a test tube for size measurements on Zetasizer 5000 (Malvern Instrument, Worcestershire, UK) with argon laser at 633.0 nm at room temperature. Liposome populations generally show narrow distribution (peak width less than 20 nm) with occasionally 1% of peak at ~300 nm by intensity (but not visible by volume or number). Mean particle size reported in manuscript represents average and standard deviation calculated from number measurements of at least 3 sizing experiments.

**Egg Phosphatidylcholine-10β-[(1-(3-pyidyl)methylpiperazyl)propyl]deoxoartemisinin Lipsome (NPm109)** **Preparation.**  Procedure was the same as that described above. 19.4μL of 10mg/mL stock of **AMPm109** in chloroform was added for making 200μL of 20mM liposome at 10:1 lipid:drug ratio.

**Loading and Release Efficiencies.** pH 7.6 buffer was made by diluting a 10X PBS buffer to 0.9X PBS, the same as used for preparing NPs. pH 6 and pH 4 buffers were made by titrating 0.95mM citric acid monohydrate with 2.67mM trisodium citrate dehydrate. Sodium chloride was then added to make buffer solutions containing 0.81% sodium chloride. The osmotic pressures of the buffers were tested to ensure that there’s no significant variation.

For loading efficiency studies, after the liposomes were made, 500μL out of 600μL of the liposome suspension were placed into a dialysis tubing (MWCO 6000-8000 Da) and dialyzed against 500mL of 0.9X PBS buffer for 16 hours at room temperature. 30μL of both dialyzed and undialyzed samples were collected in separate 5mL glass test tubes. The liposome samples were diluted to 100μL with 0.9X PBS buffer, and extracted with 1mL of spectroscopy-grade DCM by pipetting in and out 10 times with a150mm disposable glass transfer pipet. After extraction, 0.8mL of the organic layer was collected in a clean test tube. The extraction process was repeated for a total of 3 times, and the combined organic layer was dried over Na_2_SO_4_. The solvent was then removed *in vacuo*. The dried solids were redissolved in 900μL of acetonitrile (HPLC grade), divided into three 300μl portion and each portion used for the UV absorbance measurement from 200 to 400nm by DU 640 spectrophotometer (Beckman Culter, USA).

The percent loaded was calculated according to the following equation (**Eq. S1**):


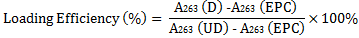


Where A_263_ (D) is th absorbance at 263nm of the dialyzed sample, A_263_ (UD) is that of the undialyzed sample, and A_263_ (EPC) is that of EPC alone. Values used for calculation for a single experiment are the average of triplicate readings.

For the release efficiency studies 150μL of 20mM dialysis-purified liposome suspensions were placed in dialysis tubing and dialyzed against 500mL of buffers of different pHs for 24 hours at room temperature. 30μL of dialyzed samples were collected afterward for UV studies with the same workup procedure as that of the loading efficiency studies.

The percent released was calculated according to **Eq.2** :


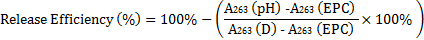


Where A_263_ (pH) is the absorbance at 263nm of the sample dialyzed for 24 hours at various pH values, A_263_ (D) is that of the dialysis-purified sample, and A_263_ (EPC) is that of EPC alone. Values used for calculation for a single experiment are the average of triplicate readings.

**MTT Assays.** In a 96 well plate was seeded ca. 2000 cells/well for BT474 or 7000cells/well for MDA-MB-231 and incubated for 18 hours at 5% CO_2_ in DMEM containing 10% FBS (Complete Medium) at 37°C. Serial dilutions of ADP stock solutions at 20mM in DMSO were made to 8 appropriate concentrations ranging from1nM to100μM, depending on the specific compound, in Complete Medium with 1% DMSO. 200μL of the compound containing medium were added to each well after removal of the original exhausted medium. NP concentrations were calculated assuming 100% loading for both formulations. Three wells were run in parallel for any given compound and concentration. The negative control was 1% DMSO containing Complete Medium and positive control employed 100μM ART dimer succinate. The cells were incubated with the drugs for 48 hours at 37°C before the medium was replaced with 90μl of fresh Complete Medium plus 10μl of MTT solution at 5mg/mL concentration and incubated further for 4 hours. At the end of incubation time, exhausted medium was gently removed and the purple formazan crystals were dissolved in 50μl of DMSO, incubated for 10 minutes before the absorbance at 570nm was read on microplate reader model 680 (Bio-Rad, California, USA).

Artemisinin Dimer Piperazine Conjugates (ADPs)

(b)

**AMPm109**

|  | **Aqueous Solubility with 5% DMSO (mM)** | | | |
| --- | --- | --- | --- | --- |
|  | pH 7.4 | | pH 6 | pH 4 |
| **ADP106** | <<0.055 | 0.082 | | 1.6 |
| **ADP107** | <<0.048 | <<0.048 | | 0.14 |
| **ADP108** | 0.25 | | 0.57 | 1.1 |
| **ADP109** | 0.034 | | 0.19 | 1.5 |
| **AMPm109** | 1.4 | | >2 | >2 |

**Table S1**. Solubility of compounds **ADP106-109**, **AMPm109** in phosphate/citrate buffer with 5% DMSO, estimated from visual turbidity test.

|  | IC50 (μM) | |
| --- | --- | --- |
|  | SKBR3 (± S.D.) | MDA-MB468 (± S.D.) |
| ADP109 | 7 ( ±2) | 2.6 (±0.4) |
| NP109 | >10 | >10 |

**Table S2**. IC50 values of **ADP109** and **NP109** on SKBR3 and MDA-MB-468 cell lines, values reported as mean and standard deviation of triplicate independent experiments.

**Reference**

1. Posner, G. H.; Paik, I. H.; Sur, S.; McRiner, A. J.; Borstnik, K.; Xie, S. J.; Shapiro, T. A., Orally active, antimalarial, anticancer, artemisinin-derived trioxane dimers with high stability and efficacy. *Journal of Medicinal Chemistry* **2003,** *46* (6), 1060-1065.

2. Chadwick, J.; Mercer, A. E.; Park, B. K.; Cosstick, R.; O'Neill, P. M., Synthesis and biological evaluation of extraordinarily potent C-10 carba artemisinin dimers against P-falciparum malaria parasites and HL-60 cancer cells. *Bioorganic & Medicinal Chemistry* **2009,** *17* (3), 1325-1338.

3. Hindley, S.; Ward, S. A.; Storr, R. C.; Searle, N. L.; Bray, P. G.; Park, B. K.; Davies, J.; O'Neill, P. M., Mechanism-based design of parasite-targeted artemisinin derivatives: Synthesis and antimalarial activity of new diamine containing analogues. *Journal of Medicinal Chemistry* **2002,** *45* (5), 1052-1063.

4. Kinman, L.; Brodie, S. J.; Tsai, C. C.; Bui, T.; Larsen, K.; Schmidt, A.; Anderson, D.; Morton, W. R.; Hu, S. L.; Ho, R. J. Y., Lipid-drug association enhanced HIV-1 protease inhibitor indinavir localization in lymphoid tissues and viral load reduction: A proof of concept study in HIV-2(287)-infected macaques. *Jaids-Journal of Acquired Immune Deficiency Syndromes* **2003,** *34* (4), 387-397.
